# Retraction Note: Alpha-ketoglutarate promotes skeletal muscle hypertrophy and protein synthesis through Akt/mTOR signaling pathways

**DOI:** 10.1038/s41598-020-72330-x

**Published:** 2020-10-28

**Authors:** Xingcai Cai, Canjun Zhu, Yaqiong Xu, Yuanyuan Jing, Yexian Yuan, Lina Wang, Songbo Wang, Xiaotong Zhu, Ping Gao, Yongliang Zhang, Qingyan Jiang, Gang Shu

**Affiliations:** 1 National Engineering Research Center for Breeding Swine Industry, College of Animal Science, South China Agricultural University, Guangzhou, 510642 Guangdong China; 2 ALLTECH-SCAU Animal Nutrition Control Research Alliance, South China Agricultural University, Guangzhou, 510642 PR China

Retraction of: *Scientific Reports* 10.1038/srep26802, published online 26 May 2016

The authors have retracted this article.

After publication concerns were raised that lanes 1 and 2 and lanes 4 and 5 in Figure 3B appear to be duplications of each other. The authors have confirmed that there are problems with the data presented in this Figure and stated that they no longer have the original data files.

All authors agree with this retraction.

